# Acidic Cannabinoids Suppress Proinflammatory Cytokine Release by Blocking Store-operated Calcium Entry

**DOI:** 10.1093/function/zqac033

**Published:** 2022-07-08

**Authors:** Malika Faouzi, Clay Wakano, Mahealani K Monteilh-Zoller, Ram P Neupane, John G Starkus, Jayanti Bhandari Neupane, Aaron J Cullen, Brandon E Johnson, Andrea Fleig, Reinhold Penner

**Affiliations:** Center for Biomedical Research, The Queen's Medical Center, Honolulu, HI 96813, USA; Cancer Biology Program, University of Hawaii Cancer Center, Honolulu, HI 96813, USA; Center for Biomedical Research, The Queen's Medical Center, Honolulu, HI 96813, USA; Cancer Biology Program, University of Hawaii Cancer Center, Honolulu, HI 96813, USA; Center for Biomedical Research, The Queen's Medical Center, Honolulu, HI 96813, USA; Center for Biomedical Research, The Queen's Medical Center, Honolulu, HI 96813, USA; Center for Biomedical Research, The Queen's Medical Center, Honolulu, HI 96813, USA; Center for Biomedical Research, The Queen's Medical Center, Honolulu, HI 96813, USA; Center for Biomedical Research, The Queen's Medical Center, Honolulu, HI 96813, USA; Center for Biomedical Research, The Queen's Medical Center, Honolulu, HI 96813, USA; Center for Biomedical Research, The Queen's Medical Center, Honolulu, HI 96813, USA; Cancer Biology Program, University of Hawaii Cancer Center, Honolulu, HI 96813, USA; Center for Biomedical Research, The Queen's Medical Center, Honolulu, HI 96813, USA; Cancer Biology Program, University of Hawaii Cancer Center, Honolulu, HI 96813, USA

**Keywords:** cannabinoids, cannabigerolic acid, calcium-release activated calcium, inflammation, calcium inhibitor, Interleukin-2

## Abstract

*Cannabis sativa* has long been known to affect numerous biological activities. Although plant extracts, purified cannabinoids, or synthetic cannabinoid analogs have shown therapeutic potential in pain, inflammation, seizure disorders, appetite stimulation, muscle spasticity, and treatment of nausea/vomiting, the underlying mechanisms of action remain ill-defined. In this study we provide the first comprehensive overview of the effects of whole-plant *Cannabis* extracts and various pure cannabinoids on store-operated calcium (Ca^2+^) entry (SOCE) in several different immune cell lines. Store-operated Ca^2+^ entry is one of the most significant Ca^2+^ influx mechanisms in immune cells, and it is critical for the activation of T lymphocytes, leading to the release of proinflammatory cytokines and mediating inflammation and T cell proliferation, key mechanisms for maintaining chronic pain. While the two major cannabinoids cannabidiol and trans-Δ^9^-tetrahydrocannabinol were largely ineffective in inhibiting SOCE, we report for the first time that several minor cannabinoids, mainly the carboxylic acid derivatives and particularly cannabigerolic acid, demonstrated high potency against SOCE by blocking calcium release-activated calcium currents. Moreover, we show that this inhibition of SOCE resulted in a decrease of nuclear factor of activated T-cells activation and Interleukin 2 production in human T lymphocytes. Taken together, these results indicate that cannabinoid-mediated inhibition of a proinflammatory target such as SOCE may at least partially explain the anti-inflammatory and analgesic effects of *Cannabis*.

## Graphical Abstract

**Graphical Abstract:**
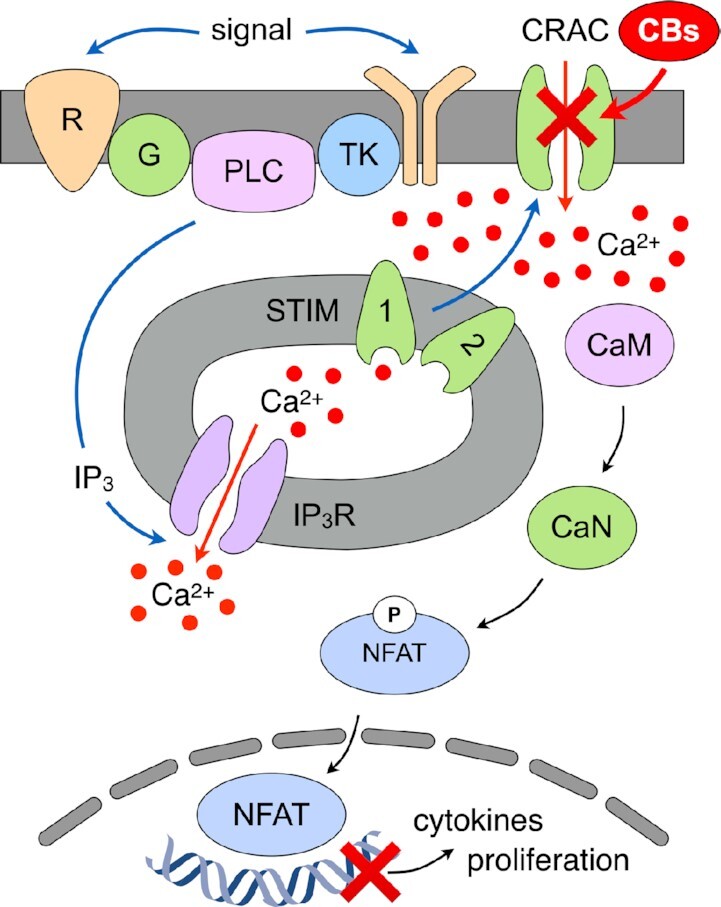
Store-Operated Calcium Entry is mediated by receptor (R) stimulation, transduced by tyrosine kinases (TK) or G proteins (G), leading to activation of phospholipase C and inositol 1,4,5 trisphosphate (IP3) production. The opening of IP3 receptors (IP3R) in the endoplasmic reticulum (ER) results in store depletion that is sensed by STIM1/2, which activate calcium (Ca2+) influx via Calcium Release-Activated Calcium (CRAC) channels. Several cannabinoids (CBx) inhibit these CRAC channels and thereby suppress signal transduction through the Calmodulin (CaM) - Calcineurin (CaN) - Nuclear Factor of Activated T cells (NFAT) pathway, which regulates cytokine release and cell proliferation.

## Introduction

*Cannabis sativa* contains a large number of chemicals, including cannabinoids, terpenes, lignans, and flavonoids. Some of these molecules have significant biological activities, and the *Cannabis* plant has been reported to have therapeutic effects for numerous medical conditions, including, neurodegenerative diseases, autoimmune diseases, pain, cancer, chemotherapy-induced nausea, and epilepsy.^1–5^ A commonality among the aforementioned medical conditions is their association with inflammation, a process that can initiate, contribute, or exacerbate their manifestation. Inflammation is a common response to tissue injury by mechanical damage or pathogen infection and is clinically manifested by swelling, heat, redness, and pain.^6,7^ Inflammation is comprised of two responses, the first is acute inflammation, occurring immediately and is nonspecific, while the other is chronic inflammation, occurring later and is specific.^8^ The acute inflammatory response initiates from damage to tissues and is characterized by the release of endogenous pro-inflammatory mediators (chemokines and cytokines) leading to changes in vasculature flow and permeability and the activation of local resident cells such as mast cells, macrophages, and monocytes, followed by polymorphonuclear leukocytes.^6^,^8–10^ Following this initial response, monocytes differentiate into macrophages, which then recruit lymphocytes.^8^ A successful response is then characterized by the replacement of proinflammatory mediators with the release of anti-inflammatory mediators, to suppress inflammation and begin with repair.^11^ If unresolved, a persistent and resolute response leads to chronic inflammation and ensuing tissue damage.

The release of proinflammatory mediators is regulated by calcium-dependent signaling mechanisms that activate several transcription factor pathways, including NFAT, nuclear factor kappa B (NF-_K_B), and cAMP response element-binding protein.^12–15^ Calcium-dependent signaling mechanisms occur through the release of Ca^2+^ from intracellular compartments as well as from influx across the plasma membrane (PM) via ion channels. However, the most significant Ca^2+^ influx mechanism in immune cells is store-operated calcium entry (SOCE), which is a critical mechanism in the activation of T cells and most immune cells.^16–19^ Store-operated calcium entry can be initiated by the activation of receptor kinases and/or G-protein coupled receptors (GPCR),^17^,^20–22^ leading to the activation of phospholipase C (PLC), which hydrolyzes phosphatidylinositol 4,5-bisphosphate (PIP_2_) into inositol 1,4,5-trisphosphate (IP_3_) and diacylglycerol (DAG). Inositol 1,4,5-trisphosphate binds to and opens the IP_3_ receptor (IP_3_R) within the endoplasmic reticulum (ER) to release Ca^2+^ into the cytosol. This depletion of Ca^2+^ in the ER is sensed by the stromal interaction proteins (STIM1, STIM2) inducing conformational changes to oligomerize, bind to, and activate the plasma membrane-resident calcium release-activated calcium (CRAC) channels (composed of Orai1,2,3 subunits).^18,19^

Previously, it was demonstrated that cannabinoids modulate calcium-permeable ion channels, in particular some members of the transient receptor potential (TRP) channels.^3,23,24^ However, their ability to modulate CRAC channels and SOCE remains entirely unknown. In this study, we comprehensively evaluated several pure cannabinoids and *Cannabis* extracts against SOCE as possible anti-inflammatory analgesics and therapeutics. Our data provide the first evidence for the inhibition of SOCE by several cannabinoids in a dose-dependent manner, with cannabigerolic acid (CBGA) having the highest potency with a submicromolar IC_50_ (IC_50_ = 530 n m). In addition, we show that incubation with CBGA inhibits T cell activation and subsequently impairs IL-2 production. Moreover, the carboxylic acid containing cannabinoids have a higher potency against SOCE than their decarboxylated counterparts, suggesting a striking structure-activity relationship (SAR). Our findings highlight a novel mechanism of action of cannabinoids and support their potential therapeutic value and the enormous implications for treating inflammatory diseases.

## Materials and Methods

### Cell Culture

Jurkat (ATCC, Manassas, VA, USA), Jurkat NFAT (BPS Biosciences, San Diego, CA, USA), THP-1 NF_K_B (BPS Biosciences), and U937 (ATCC) cell lines were maintained in RPMI 1640 medium (Clontech, San Jose, CA, USA) supplemented with 10% fetal bovine serum (Gibco, Waltham, MA, USA). RBL2H3 (ATCC) and HEK293 (ATCC) cell lines were maintained in DMEM medium (Clontech) supplemented with 10% fetal bovine serum. The Luva cell line (Kerafast, Boston, MA, USA) was maintained in StemPro-34 SFM medium supplemented with StemPro-34 Nutrient supplement and 2 m m L-glutamine (Gibco). All cells were cultured at 37°C, 5% CO_2_, and 95% humidity.

### Fluorescence Imaging of SOCE Bioassay

The activity of *Cannabis* extracts and pure cannabinoids were examined using a high-throughput kinetic plate reader (Hamamatsu FDSS 7000EX). Suspension cell lines (Jurkat, Jurkat NFAT, THP-1 NF_K_B, and U937) were incubated with Fura2-AM (ThermoFisher, Waltham, MA, USA) loading medium at 37°C, 5% CO_2_, and 95% humidity for 1 h, spun down, washed once with bath solution, and plated in black 96-well μclear, flat-bottom plates (Greiner Bio-One, Munroe, NC, USA) at 10^5^ cells/well the day of Ca^2+^ imaging. Adherent cell lines (RBL2H3, HEK293, and Luva) were plated overnight in black 96-well μclear, flat-bottom plates at 6 × 10^4^ cells/well. Poly-l-lysine (Sigma-Aldrich, St. Louis, MO, USA) coating of the plates is used for HEK293 cells due to their semi-adherent nature. The following day adherent cells were incubated with Fura2-AM loading medium at 37°C, 5% CO_2_, and 95% humidity for 1 h and washed twice with bath solution before Ca^2+^ imaging. Fura2-AM loading medium contained, RPMI 1640 or DMEM complete medium supplemented with 2 μm Fura2-AM, 1X Powerload (ThermoFisher), and 1X Probenecid (ThermoFisher). Bath solution contained (in m m): 140 NaCl, 2.8 KCl, 2 MgCl_2_, 1 CaCl_2_, 10 HEPES, and 11 glucose (pH 7.2, 300 mOsm). Fura2-AM ratiometric data were acquired at a rate of 1 Hz and normalized to a gadolinium chloride (Gd^3+^) control. SOCE bioassay protocol was as follows: baseline levels were acquired for 60 s followed by extract or pure cannabinoid addition. At 240 s, 1 µm thapsigargin (Tg) (ThermoFisher) was applied. At 600 s, 2 m m ethylene glycol-bis(β-aminoethyl ether)-N,N,N’,N’-tetraacetic acid (EGTA) (ThermoFisher) was applied to chelate all external calcium. To assess SOCE response, areas under the curve (AUC) obtained from 240–600 s were calculated using Igor Pro (WaveMetrics, Lake Oswego, OR, USA) and corrected by subtracting AUC for gadolinium response.

### Whole-Cell Patch-Clamp Recording

Jurkat NFAT cells were plated on poly-L-lysine coated coverslips, transferred to a patch chamber dish, and bathed in 2.5 mL of external Ringer's solution. Ringer's solution contained (in m m): 140 NaCl, 2.8 KCl, 2 MgCl_2_, 10 HEPES-NaOH, 10 CaCl_2_, and 11 glucose; pH 7.2, 300–330 mOsm. Electrophysiological experiments were performed in the whole-cell configuration of the patch-clamp technique. Intracellular solutions contained (in m m): 140 L-(+)-glutamic acid, titrated with cesium hydroxide, 8 NaCl, 1 MgCl_2_,10 Cs-BAPTA, 10 HEPES-CsOH, and 0.05 D-myo-Inositol 1,4,5-tri-phosphate trisodium salt (Sigma-Aldrich); pH 7.2, 300–330 mOsm. Cells were held at 0 mV and given a voltage ramp protocol from −140  to +100 mV for 50 ms, every other second. Currents obtained at −120 mV were analyzed using Igor Pro.

### HPLC Analysis

The cannabinoids were identified and quantified by high pressure liquid chromatography (HPLC) (Ultimate 3000, ThermoFisher) and photodiode (DAD-3000, ThermoFisher) array detection. Certified reference materials purchased from Cayman Chemical Company (Ann-Arbor, MI, USA) and Sigma-Aldrich were used to prepare calibration curves. Samples were injected in 5 μL volumes and eluted through a Raptor ARC-18 analytical column (Restek, Bellefonte, PA, USA) with 77% acetonitrile (buffered with 0.1% v/v formic acid) and 23% water (buffered with 5 m m ammonium formate and 0.1% v/v formic acid). Flow rate was set to 0.95 mL/min, the column compartment was maintained at 25°C and the UV-Vis signals were recorded using a DAD-3000 photodiode array detector. Calibrations and quantifications were based on absorbances measured at 228 nm.

### Size Exclusion Chromatography

Sephadex G-25 powder (fine) was obtained from Cytiva (Marlborough, MA, USA) and soaked in distilled water for 4 h. The slurry was loaded into a spin column (EndotoxinOUT, G-Biosciences, St. Louis, MO, USA) and packed to a resin volume of 3 mL. A 0.5 mL sample of 30 μm CBGA in Ringer's solution with or without 3 mg/mL bovine serum albumin (BSA) (Sigma-Aldrich) was loaded onto the column and eluted with Ringer's solution using gravity. During each size exclusion chromatography (SEC) experiment, 15 fractions were collected in 1 mL volumes and each fraction was analyzed for CBGA content by HPLC.

### Isobolographic Analysis

Isoboles for each combination of CBGA with another were generated by calculating the inhibitory concentrations (IC): IC_10_, IC_20_, IC_30_, IC_40_, and IC_50_ of each pair as derived from the relevant dose–response curves such that the sum of the effects of the two cannabinoids would result in a predicted 50% inhibition of SOCE in the Jurkat NFAT reporter cells provided simple additivity of the two compound combinations. For example, in the absence of any interactions, IC_10_ of CBGA (178 n m) combined with IC_40_ of CBD (5.29 μm) would be expected to inhibit SOCE by 50%. The cannabinoid pairs were screened for any potential synergistic or inhibitory effects between CBGA and other cannabinoids.

### NFAT Reporter Assay

Jurkat NFAT reporter cell line (BPS Bioscience, San Diego, CA, USA) was maintained in RPMI 1640 medium supplemented with 10% FBS. This cell line was stably transfected with a firefly luciferase gene under the control of the NFAT transcriptional response element and has been validated for response to thapsigargin (ThermoFisher), ionomycin, phorbol 12-myristate 13-acetate (PMA) (Sigma-Aldrich) as well as a variety of T-cell receptor (TCR) activators, including anti-CD3 antibody (ThermoFisher). To measure the NFAT activity, we harvested cells from culture and seeded them at a density of 4 × 10^4^ cells/well into a 96-well clear-bottom microplate in 50 µL culture medium. Cells that were assayed in various concentrations of FBS were first washed in serum-free medium before resuspending them in medium containing the desired concentration of FBS. After plating, cells were pretreated with the cannabinoids at the desired final concentrations for 30 min, then stimulated with a combination of 1 µm ionomycin and 20 ng/mL PMA for 5 h. To measure the luciferase activity, we added 1:1 volume of the One-Step luciferase reagent (BPS Bioscience) and incubated plates for an additional 15 min before measuring the NFAT activity using a microplate reader (VICTOR^®^ Nivo^™^ Multimode, PerkinElmer, Waltham, MA, USA). Each run included untreated/unstimulated cells and vehicle-treated/unstimulated cells as negative controls. All conditions were tested in triplicate within each run and repeated in four or five independent experiments.

### IL-2 Release Assay

Jurkat cells were plated in 24-well plates (Greiner Bio-One, Munroe, NC, USA) at a density of 10^6^ cells/well in 500 µL medium. After plating, cells were treated with CBGA at final concentrations ranging from 0.1 to 100 µm, then incubated for 30 min. Following the CBGA pretreatment, cells were stimulated with a combination of 1 µm ionomycin and 20 ng/mL PMA for 24 h. Cells were then pelleted and the supernatants were transferred into microcentrifuge tubes and assayed for IL-2 release using an enzyme-linked immunosorbent assay (ELISA) (IL-2 Human Instant ELISA^TM^ kit, Thermofisher). IL-2 release following the exposure to CBGA was compared to the stimulated and untreated cells as well as to the stimulated cells treated with the vehicle, dimethyl sulfoxide (DMSO) (Sigma-Aldrich).

### Viability Assay

Jurkat cells were plated in 24-well plates at a density of 2 × 10^5^ cells/well in 500 µL medium. After plating, cells were treated with CBGA at final concentrations ranging from 0.1 to 100 µm, then incubated for 24 h. Cell viability was measured at day 0 pretreatment and 24 h post-treatment using a tetrazolium salt assay [3-(4,5-dimethylthiazol-2-yl)-2,5-diphenyltetrazolium bromide (MTT) Sigma-Aldrich]. MTT was prepared in culture medium and added to the cells at a final concentration of 0.5 mg/mL. Cells were then incubated at 37°C for 1 h to allow the metabolically active cells to reduce the dye into purple formazan. Following the incubation, we pelleted the cells, removed the supernatants, and added DMSO to dissolve the formazan crystals. Absorbance was then measured at 570 nm on a microplate reader (VICTOR^®^ Nivo^™^ Multimode, PerkinElmer).

### Statistical Analyses

Values are expressed as mean ± SEM. Statistical analysis of the data was performed using ANOVA and a post-hoc test (The Tukey test) as appropriate. Differences were considered significant at *P*< .05.

## Results

### Effects of Whole-Plant *Cannabis* Extracts on Store-Operated Ca^2^^±^ Signaling

To determine whether *Cannabis*-derived cannabinoids impacted SOCE, we initially screened two whole-plant *Cannabis* extracts obtained from the National Institute of Drug Abuse (NIDA) against our SOCE high-throughput screening (HTS) bioassay. These prototypical NIDA-sourced extracts contained naturally occurring minor cannabinoids in addition to either high levels of CBD or THC (CBD and THC extracts, respectively). Concentrations of CBD and THC were provided by NIDA's Certificate of Analysis and it was determined that 25 μg/mL of the CBD-rich extract contained the equivalent of 47 μm CBD and 2 μm THC, whereas the THC-rich extract contained 17 μm THC and 1 μm CBD.

**Figure 1.**
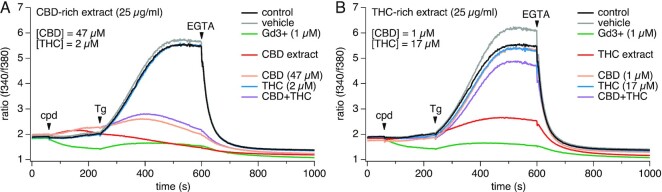
Effect of *Cannabis* extracts and pure CBD and THC on SOCE. *Cannabis* extracts (CBD- and THC-rich extracts, respectively), pure CBD and pure THC were screened against SOCE in Jurkat T cells. Baseline levels were acquired for 60 s followed by extract or pure cannabinoid addition. To activate SOCE, 1 µm Tg was applied at 240 s and 2 m m EGTA was applied at 600 s to chelate all external calcium. (A) CBD-rich extract (25 μg/mL) and equivalent concentrations of CBD (47 μm) or THC (2 μm) or both were screened against SOCE. (B) THC-rich extract (25 μg/mL) and equivalent concentrations of THC (17 μm) and CBD (1 μm) were screened against SOCE. All experiments were repeated in three independent runs. The traces represent averaged data from *n* = 3 replicates.

Jurkat T cells were used to screen for modulation of SOCE, as this is a well-established and validated model for studying this mechanism. Jurkat cells were stimulated by thapsigargin to cause intracellular Ca^2+^ store depletion and thereby activating Ca^2+^ entry through CRAC channels. Interestingly, both CBD and THC extracts at 25 μg/mL significantly inhibited SOCE, causing full block in the case of the CBD-rich extract (Figure 1A) and around 70% block in the case of the THC-rich extract (Figure 1B). To elucidate whether CBD, THC, or a combination of both were responsible for the observed effects, we screened the equivalent concentrations of pure CBD, THC, and the combination of both against SOCE. Exposing cells to pure CBD and THC at concentrations equivalent to those of the CBD extract shows that THC at 2 µm had no effect while CBD at 47 µm accounted for most of the inhibitory effect observed with the CBD-rich extract (Figure 1A). Interestingly, the addition of 17 μm pure THC alone, 1 μm pure CBD alone or a combination of both showed a minimal to no inhibitory effects on SOCE (Figure 1B), indicating that some other constituent in this extract must account for the 70% inhibition observed with the THC-rich extract. Our results suggest that these whole-plant extracts may contain minor phytochemicals that act alone or in synergy with the major cannabinoids to inhibit SOCE.

### Characterization of the Efficacy and Potency of Pure Cannabinoids on SOCE

**Figure 2.**
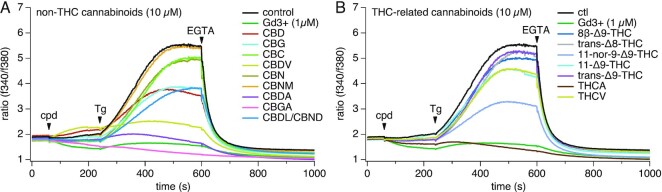
Effect of pure cannabinoids on SOCE. Pure cannabinoids (10 µm) were screened against SOCE in Jurkat T cells. Baseline levels were acquired for 60 s followed by extract or pure cannabinoid addition. To activate SOCE, 1 µm Tg was applied at 240 s and 2 m m EGTA was applied at 600 s to chelate all external calcium. (A) Non-THC cannabinoids (10 µm) were screened against SOCE. (B) THC-related cannabinoids (10 µm) were screened against SOCE. All experiments were repeated in three independent runs. The traces represent averaged data from *n* = 3 replicates.

We next tested both the major and minor pure cannabinoids as well as some metabolites to assess their inhibitory effect on SOCE. Initially, we screened these cannabinoids at 10 μm in close analogy to the experiments described previously. Surprisingly, several minor cannabinoids, such as CBGA, cannabidiolic acid (CBDA), cannabidivarin (CBDV), and tetrahydrocannabinolic acid (THCA) showed a higher inhibitory effect of SOCE than the major cannabinoids CBD and THC (Figure 2A and B). CBGA emerged as the most potent non-THC cannabinoids tested (Figure 2A), slightly more potent than THCA from the THC-related group (Figure 2B). Both induced a comparable effect to 1 µm Gd^3+^, which we used as a positive control. Interestingly, while trans-∆9-THC (commonly known as THC) was essentially inactive, one of its known metabolites, 11-nor-9-carboxy-Δ9-tetrahydrocannabinol (11-nor-9-carboxy-THC) showed strong inhibition of SOCE at 10 µm. Our results demonstrate that, several minor cannabinoids appear to be more potent in suppressing SOCE than either THC or CBD.

### Concentration–Response Effects of Pure Cannabinoids on SOCE

**Figure 3.**
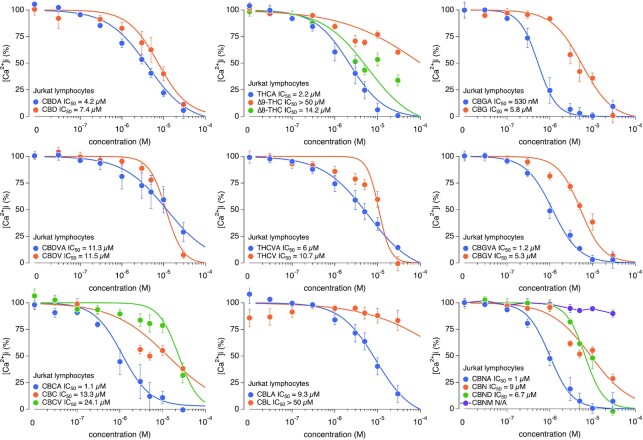
Pure cannabinoids concentration–response effects on SOCE. Pure cannabinoids concentration–response relationships ranging from 30 n m to 30 µm were assayed against SOCE. Corresponding IC_50_ values for each cannabinoid are indicated in the graph legends. All data points represent normalized mean ± SEM of *n* = 3 independent replicates.

Since we observed several cannabinoids with inhibitory effects on SOCE at 10 μm, we established full dose–response curves for all cannabinoids to determine the relative potency for each of them. Figure 3 shows the eight-point concentration–response curves, ranging from 30 n m to 30 µm, for 22 pure cannabinoids screened against SOCE in a Jurkat NFAT reporter cell line. The IC_50_ derived from least-squares fits to the data values ranging from as low as 530 n m (CBGA) to >50 µm [cannabinol methyl ether (CBNM), cannabicyclol (CBL), and THC]. Strikingly, our results unveiled a clear pharmacological distinction between the acidic and decarboxylated cannabinoids in that generally, the carboxylic acid-containing cannabinoids appeared to have significantly greater potencies in inhibiting SOCE compared to their decarboxylated counterparts and other structurally related compounds, indicating a possible SAR associated with this functional group. These results also confirm our initial observation from the single-dose screening that CBGA is indeed the most potent of all the cannabinoids tested. We then set out to further investigate this compound and determine CBGA's mechanism of action in T cell calcium signaling and activation.

### CBGA Blocks CRAC Current in a Dose-Dependent Manner

**Figure 4.**
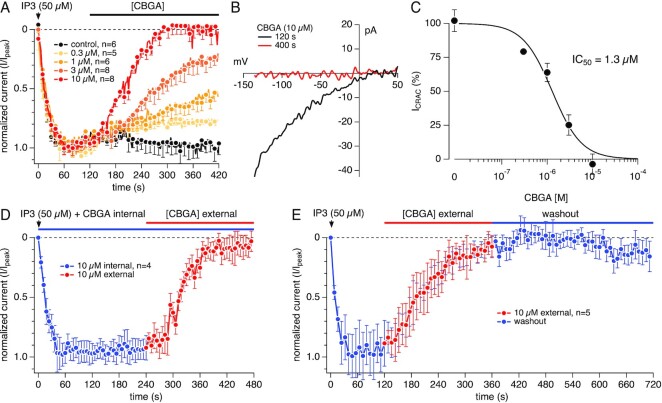
Concentration-dependent inhibition of CRAC current by CBGA. (A) Time course of CRAC currents recorded in Jurkat T cell in whole-cell patch-clamp experiments. 50 µm IP_3_ in the recording pipette was utilized to elicit the active calcium depletion from the ER. The resulting CRAC currents were allowed to reach maximal activation prior to extracellular application of CBGA at various concentrations. Current amplitudes were measured at −120 mV for each voltage ramp and were normalized to the control at 120 s. *n* represents the number of cells patched for each condition. (B) Representative current versus voltage (I/V) relationship of I_CRAC_. Single current traces taken from a control cell before (black trace) and after (red trace) exposure to 10 µm CBGA. (C) Concentration–response curve of CBGA for I_CRAC_. Data points for the curve were obtained from the normalized currents in panel A at 400 s. (D) Time course of IP_3_-induced CRAC currents recorded in Jurkat T cell in whole-cell patch-clamp experiments. To investigate the effect of CBGA intracellularly, 10 µm CBGA was added in the recording pipette containing IP_3_. The resulting CRAC currents were allowed to be exposed to internal CBGA for 240 s prior to extracellular application of 10 µm CBGA (*n* = 4). (E) Time course of IP_3_-induced CRAC currents. The resulting CRAC currents were allowed to reach maximal activation prior to extracellular application of 10 µm CBGA. After reaching full inhibition, CBGA was then washed out with external application of the bath solution (*n* = 5).

Jurkat cells have been shown to express CRAC channels.^25,26^ To examine whether CBGA affects SOCE through inhibition of these channels, we used whole-cell patch-clamp electrophysiological recordings to measure CRAC currents (I_CRAC_) in Jurkat NFAT cells. This was achieved by perfusing the cytosol with 50 µm IP_3_ to deplete Ca^2+^ stores via the IP_3_R and trigger the CRAC current I_CRAC_. Figure 4A shows the kinetics of the elicited I_CRAC_ activation and its inhibition by CBGA as measured at −120 mV. CBGA blocks I_CRAC_ in a dose-dependent manner, with full inhibition achieved by 10 µm (Figure 4A). Figure 4B illustrates the current versus voltage (I/V) relationship of I_CRAC_ taken at the peak activation (120 s) and after steady-state inhibition by 10 µm CBGA (400 s). The black trace shows the signature inwardly rectifying current/voltage (I/V) relationship of I_CRAC_ (Figure 4B). The red trace demonstrates that the application of CBGA (10 µm) completely inhibited I_CRAC_ (Figure 4B). Figure 4C illustrates the concentration–response curve of CBGA-mediated inhibition of CRAC currents, yielding a calculated IC_50_ of 1.3 µm. Our results confirm that CBGA modulates SOCE in T cells through inhibition of CRAC channels.

The internal application of CBGA resulted in no block of the currents (Figure 4D), suggesting that CBGA acts at an external or plasma-membrane delimited site on the CRAC channels. Figure 4 also shows that the effect of CBGA is persistent and poorly reversible, at least for a 6 min period of rigorous washout in whole-cell recordings (Figure 4E). This would imply that relatively low doses or a less frequent dosing regimen might be sufficient to block I_CRAC_ in vivo. Taken together, these findings not only demonstrate a novel mechanism of action of CBGA, and possibly other cannabinoids, but also brings to light a novel potent inhibitor of CRAC channels, which are highly sought-after pharmacological targets in drug development against several inflammatory disorders.

### Cannabinoids Fail to Inhibit NFAT Activation in Standard Culture Conditions

Various signaling pathways, including the inositol triphosphate (IP_3_)–Ca^2+^–NFAT pathway are involved in TCR signaling and T cell activation^27^ and CRAC channels constitute an essential upstream component of the NFAT pathway.^28^ Given our results of CBGA blocking SOCE and I_CRAC_, we set out to determine whether CBGA affects NFAT, an important calcium-dependent transcription factor that regulates subsequent production and release of IL-2. Using Jurkat T cells expressing an NFAT reporter gene, we performed a single-dose screening of our panel of pure cannabinoids on NFAT activation. Jurkat cells were activated with PMA and ionomycin, a widely used in vitro stimulus to activate TCR downstream pathways without the need of a TCR agonist. PMA is a small organic compound that mimics DAG in activating protein kinase C (PKC), triggering the PKC-IkappaB kinase (IKK)-NF-κB pathway. Ionomycin is a Ca^2+^ ionophore that triggers Ca^2+^ release, hence activating the IP_3_-SOCE-NFAT pathway.

**Figure 5.**
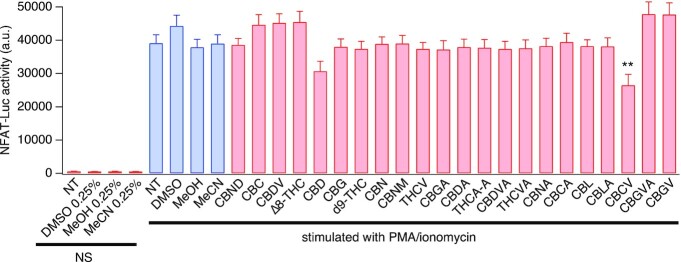
Effect of cannabinoids on NFAT activity in standard culture medium. Pure cannabinoids were tested at 5 µm against NFAT activity. Jurkat T cells expressing NFAT reporter gene were pretreated with the cannabinoids before stimulation using 1 µm ionomycin and 20 ng/mL PMA. Bioluminescence emitted by NFAT-mediated luciferase expression was measured in arbitrary units 5 h poststimulation. All data points represent mean ± SEM of 3 independent runs (*N* = 3), with each run including *n* = 3 replicates per condition. *P< .05, **P< .01. Key: NT = nontreated cells, NS = nonstimulated, MeOH = methanol, MeCN = acetonitrile, DMSO = dimethylsulfoxide.

We tested the cannabinoids at 5 µm, a concentration that, based on the dose–response behavior of all compounds, would allow us to differentiate between potent, less potent, and inactive cannabinoids. Surprisingly, and despite their activity against SOCE, none of the cannabinoids showed a significant inhibition of NFAT, including our lead compound CBGA (Figure 5). We observed a minor effect with CBD and CBCV (21.6% and 32.3% block, respectively). A possible explanation for this lack of NFAT suppression could be that the cannabinoids are either metabolized or sequestered by tissue culture medium ingredients, for example, serum. Previous reports demonstrated that cannabinoids display a higher potency in vitro at lower serum concentrations, suggesting that serum proteins render cannabinoids biologically inactive.^29,30^

### Stability of CBGA Under Standard Tissue Culture Conditions

**Figure 6.**
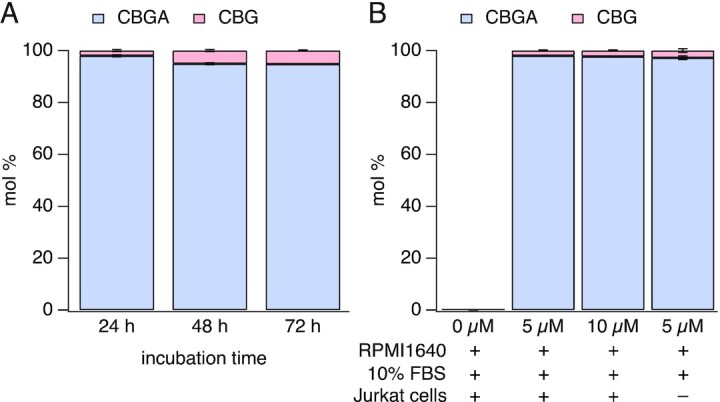
HPLC analysis of CBGA and CBG present in culture medium. HPLC was used to analyze for degradation or decarboxylation of CBGA in standard culture conditions. (A) Analysis of the presence of CBGA and CBG in culture medium (RPMI 1640) with no serum (*n* = 3). (B) Analysis of CBGA and CBG contents in standard culture medium (RPMI 1640 + 10% FBS), in the presence or absence of Jurkat T cells (*n* = 3). In both cases, CBGA was incubated at 37°C, 5% CO_2_, and 95% humidity to mimic the tissue culture conditions. Values are averaged mol % ± SEM.

To investigate whether CBGA is metabolized in tissue culture medium, we analyzed CBGA concentrations (5 µm and 10 µm) over a period of 24, 48, and 72 h in serum-free medium (RPMI1640 only) by HPLC. HPLC analysis showed no significant decrease in the concentration of CBGA over time. Figure 6A shows only the results for 5 µm CBGA but similar results were obtained for 10 µm as well. The minor loss of CBGA may be attributed to decarboxylation of CBGA to cannabigerol (CBG) (only up to 5 mol % after 72 h) (Figure 6A). A similar experiment was carried out in culture medium supplemented with 10% (v/v) FBS following incubation for 24 h. The experiment yielded similar results, with very little decarboxylation, irrespective of the presence or absence of Jurkat T cells during incubation (Figure 6B). The apparent stability of CBGA in culture conditions suggests that the lack of response in the NFAT activation assay is not due to chemical metabolism or breakdown of the compound.

### CBGA Activity Decreases as Serum Concentration Levels Increase

**Figure 7.**
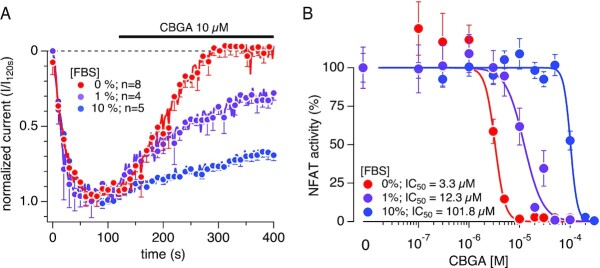
CBGA inhibitory potency is decreased by increasing serum concentrations. Serum-dependent effects of CBGA on I_CRAC_ and NFAT activity in the presence of 0%, 1%, and 10% FBS. (A) Time course of CRAC currents recorded in Jurkat T cell in whole-cell patch-clamp experiments. Bath solution was supplemented with 0%, 1%, or 10% FBS (0% serum data is the same as in Figure 4A). To elicit the active calcium depletion from the ER, 50 µm IP_3_ in the recording pipette was utilized. The resulting CRAC currents were allowed to reach maximal activation prior to extracellular application of CBGA at various concentrations. Current amplitudes were measured at −120 mV for each voltage ramp and were normalized to the control at 120 s. *n* represents the number of cells patched for each condition. (B) Concentration–response effects of CBGA on NFAT activity in the presence of 0%, 1%, and 10% FBS. A 10- and 12-point concentration-response curves, ranging from 100 n m to 100 µm and 100 n m to 300 µm, respectively, were established. The 12-point d/r was used for 10% FBS condition due to the weak activity of CBGA in the ten-point concentration range under this condition. Values are average ± SEM of 12–15 replicates, normalized to the control (cells treated with PMA + Ionomycin only).

It is well known that serum albumins found in FBS possess intrinsic fluorescence that is excited within the UV spectrum due to their tryptophan residues. We hence opted out of testing CBGA in the above-described SOCE assay, which uses a UV-excitable dye (Fura2-AM). Instead, we investigated the impact of FBS presence on CBGA bioavailability directly on CRAC channel activity and on the downstream NFAT activity. We first tested CBGA against I_CRAC_ in the absence or presence of 1% or 10% FBS in the bath solution (Ringer's solution) (Figure 7A). We screened CBGA at 10 µm, which showed a full block of I_CRAC_ in the absence of serum (Figure 4A). We observed that the addition of FBS to the bath solution did not affect the activation rate or amplitude of I_CRAC_, but prevented CBGA from inhibiting the channels. This loss of activity was correlated with the increase of FBS concentration. We then tested CBGA on NFAT activity by changing the FBS content in the culture medium. Figure 7B shows that CBGA inhibits NFAT activity in a dose-dependent manner with an IC_50_ of 3.3 µm in the absence of FBS. Increasing FBS in the culture medium, however, resulted in a dose-dependent shift of CBGA's IC_50_. We observed a 3.7-fold and a 30-fold increase of the IC_50_ values in the presence of 1% FBS and 10% FBS, respectively (Figure 7B). Taken together, our results confirm that serum proteins may play a role in suppressing the inhibitory effect of CBGA, and possibly other cannabinoids, when complete standard medium is used in the in vitro assays.

### CBGA Binds to Serum Albumins

**Figure 8.**
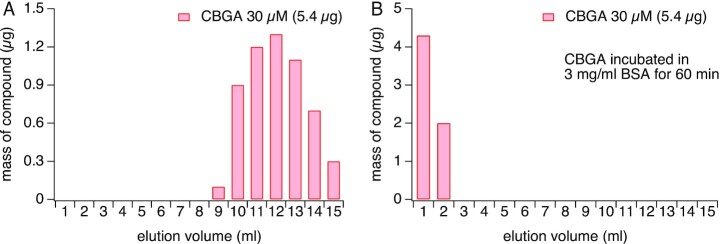
Size exclusion chromatography analysis of CBGA. (A) Analysis of fractions collected when CBGA was eluted through a Sephadex G-25 size exclusion column with Ringer's solution shows that CBGA elutes after fraction 9. (B) CBGA, when incubated with BSA and subjected to an identical SEC experiment, elutes early in fractions 1 and 2.

We next sought to investigate whether the inactivity of CBGA in the assay was indeed due to sequestration of the compound by serum contained in the culture medium. As albumins are known to transport peptides, fatty acids, and drug molecules in vivo, we turned our attention to BSA, which is the most abundant protein in FBS. By performing SEC on the Sephadex G-25 column, followed by HPLC analysis of collected fractions, we expect CBGA (360.5 g/mol) to elute off the column in the later fractions due to the slower migration of smaller molecular weight compounds. Conversely, we expect that BSA (66 430.3 g/mol) will elute off the column in earlier fractions due to its higher molecular weight and migrate through the column faster. As expected, we observed that CBGA alone elutes off the column in the later fractions (9–15) (Figure 8A). We next incubated 30 μm CBGA with 3 mg/mL BSA (average equivalent of BSA content in 10% FBS) and performed SEC. If CBGA binds to BSA we would predict that CBGA would elute off the column in the earlier fractions with BSA. Indeed, we observed that CBGA elutes off the column in the earlier fractions, suggesting a strong affinity of CBGA to BSA (Figure 8B). This binding of CBGA and BSA may explain the lack of activity of CBGA in assays containing 10% FBS.

### CBGA Inhibits IL-2 Production in Jurkat T Cells

**Figure 9.**
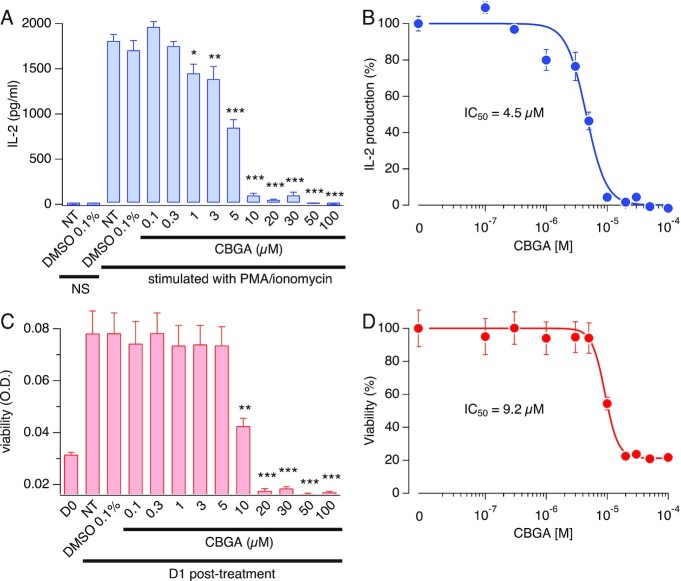
CBGA attenuates IL-2 production in Jurkat T cells. Evaluation of IL-2 levels and cell viability following treatment with CBGA. (A) Jurkat T cells were pretreated with CBGA at doses ranging from 0.1 µm to 100 µm for 30 min then stimulated with 1 µm ionomycin and 20 ng/mL PMA. Supernatants were harvested at 24 h post-treatment and IL-2 concentrations were measured by ELISA. All data points represent mean ± SEM of four independent runs (*N* = 4), each run containing a triplicate per condition. *P < .05, ***P< .001. Key: NT = nontreated cells, NS = nonstimulated. (B) Concentration–response fit of the effect of CBGA on IL-2. Data points are IL-2 concentrations obtained in A and normalized to the control (stimulated cells in the absence of CBGA). (C) Effect of CBGA on Jurkat T cells viability over a period of 24 h. Viability was measured at day 0 (D0) when cells were seeded, and at 24 h post-treatment (D1: day 1) with different doses of CBGA. All data points represent mean ± SEM of four independent runs (*N* = 4), each run containing a triplicate per condition. ***P< .001. Key: D0: day 0 prior to treatment, D1: day 1 post-treatment. NT = nontreated cells. (D) Concentration–response fit of the effect of CBGA on cell viability. Data points are optical density (O.D.) values obtained in C and normalized to the nontreated cells at day 1.

Since NFAT activation is a critical signaling mechanism that translates into IL-2 production by activated T cells, we investigated the ability of CBGA to suppress IL-2 production. Ideally, FBS-free medium would give us the dose–response effect of free CBGA. However, we used culture medium supplemented with 1% FBS, which supports reasonable bioavailability of CBGA while providing a source of nutrients to the cells and avoids starvation-induced cell stress. As shown in Figure 9A and B, Jurkat cells do not produce any IL-2 within 24 h of incubation in culture medium. PMA and ionomycin administration induced a significant amount of IL-2 that was not affected by 0.1% DMSO, which is the equivalent percent of DMSO present in our highest tested concentration of CBGA (100 µm). Under these conditions, CBGA induced a steep dose-dependent inhibition of IL-2, starting at 1 µm and reaching its maximum at 10 µm, yielding an IC_50_ of 4.5 µm. While running the IL-2 assay, we observed cell death in the high concentrations of CBGA (>10 µm), which we could not attribute to CBGA alone or to the combination of CBGA with PMA and ionomycin. To confirm these results, we assessed CBGA effects on Jurkat cell viability in the absence of any stimulation with PMA and ionomycin. In Figure 9C and D, Jurkat cells were subjected to various CBGA concentrations for 24 h, similar to the IL-2 assay. We found that CBGA exhibits cytotoxic effects at the higher concentrations (>10 µm). This effect may be specific to the continuous exposure of high cannabinoid levels at low serum levels in our in vitro assays and not apply to in vivo effects of CBGA. Previous studies have used CBGA up to 100 mg/kg in mouse and have not reported any toxicity.^31,32^ Taken together, our results suggest that CBGA inhibits the production of IL-2 at low concentrations (0–5 µm) without cytotoxic effects, concomitant with the observed effects on SOCE and NFAT. However, higher doses of CBGA can induce cell death that may be related to calcium deprivation and/or other calcium-independent cell death mechanisms.

### Combinatory Effects of CBGA with Other Cannabinoids

**Figure 10.**
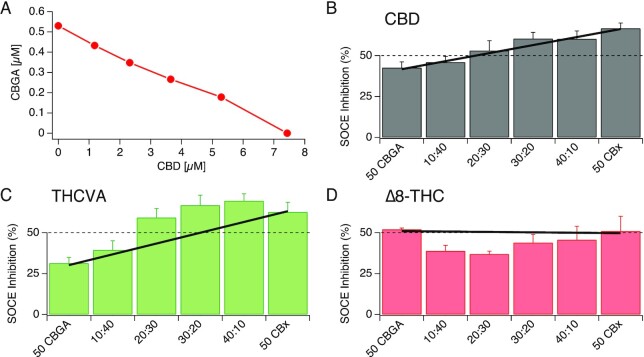
Isobolographic analysis of CBGA and other cannabinoids against SOCE. (A) An isobole showing dose pairs of CBGA and CBD that are expected to inhibit SOCE by 50%. The y- and the x-intercepts represent the IC_50_ values of CBGA and CBD, respectively. (B) Inhibition of SOCE observed when Jurkat NFAT cells were treated with concentration pairs of CBGA and CBD. The dashed line depicts the expected 50% inhibition of SOCE at each dose pair. The bold line connects the actual inhibition observed when treated separately with CBGA and the other cannabinoid (CBD) at individual IC_50_. The bars represent the observed inhibition when treated with various concentration pairs of the two compounds. Here, the bars fall on the bold line, indicating that the two compounds follow a simple additivity behavior. (C) Dose pairs of CBGA and THCVA show deviation from expected inhibition. The green bars above the bold line suggest supra-additive (synergistic) effects between the two compounds. (D) Concentration pairs of CBGA and Δ8-THC show deviation in the opposite direction, suggesting subadditive (inhibitory) effects.

To assess any potential combinatorial, or “entourage” effects between CBGA and other cannabinoids, we performed an isobolographic analysis by pairing CBGA with other cannabinoids at various concentrations and screened them in our SOCE assay (see “Materials and Methods”). The dose pairs expected to inhibit SOCE by 50% through simple additivity, were determined from the dose response curves of individual cannabinoids (eg, see Figure 10A). Any deviation from the simple additivity behavior would suggest a supra-additive (synergistic) or a subadditive (inhibitory) effect of the paired compounds. Examples of compound pairs showing simple additivity, synergistic or noncooperative effects are shown in Figure 10B, C, and D, respectively.

Prior to executing the experiment, the expected SOCE inhibition for all these dose pairs is 50%, which is depicted by a dashed line (Figure 10). However, the responses of individual compounds when dosed at their IC_50_ values did not always show the expected 50% inhibition of SOCE, owing to variabilities in experiments. Therefore, the corrected expected effects are shown by a bold line, which connects the inhibitions observed when CBGA and the other cannabinoid were exposed to their IC_50_ concentrations (Figure 10B, C, and D). For each assay plate, the response elicited by each dose pair was then measured against this bold line to determine if there were any deviations from the simple additive behavior. The pair of CBGA and CBD, for example, shows no deviation from the corrected expected effects and hence is categorized as showing simple additive behavior (Figure 10B). Certain dose pairs such as CBGA and tetrahydrocannabivarin (THCVA), however, show greater inhibition of SOCE than indicated by the bold line; therefore, these dose pairs exhibit some synergistic effects (Figure 10C). On the other hand, certain dose pairs such as CBGA and delta-8-tetrahydrocannabinol (Δ^8^-THC) show smaller than expected inhibitory effects (Figure 10D). Similarly, to what is displayed in Figure 10, we tested all combinations of CBGA with different cannabinoids (data not shown). Overall, our results show that CBGA potency can be modulated by other cannabinoids; however, we observed only slight supra-or subadditive effects of no more than a 10% increase or decrease in CBGA potency, suggesting moderate entourage effects under these conditions.

### Entourage Effect of the Cannabinoids Across Cell Lines

**Figure 11.**
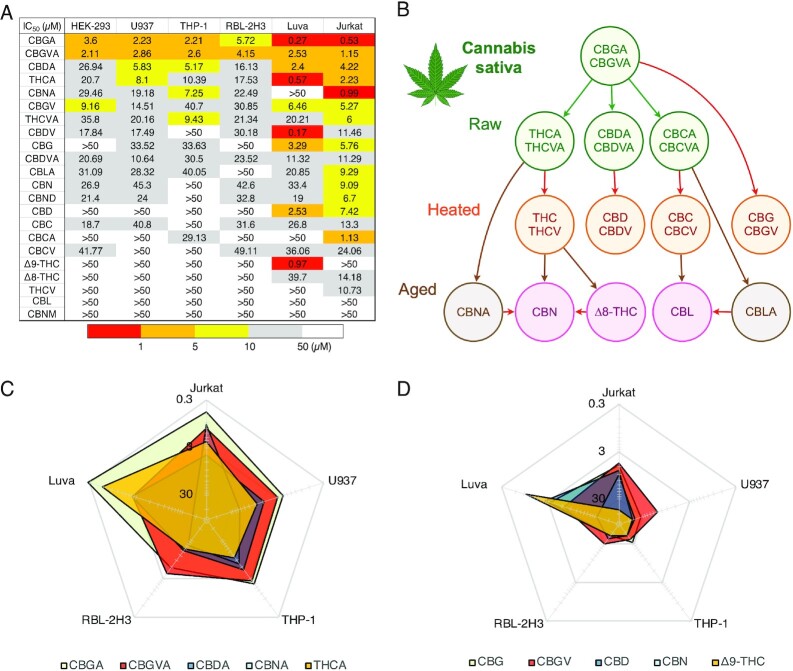
Differential potency of cannabinoids in modulating SOCE across various immune cells. (A) Heat map demonstrating IC_50_ values of cannabinoids against SOCE across 5 immune cell lines and 1 nonimmunological cell line. Red colored boxes indicate higher potency and white boxes indicate lower potency of SOCE inhibition (see table legend). (B) Biosynthetic pathway of cannabinoids in the *Cannabis* plant. Arrows indicate the direction of metabolism from precursor cannabinoids. (C) IC_50_ values of the top 5 most potent cannabinoids represented in a radar chart. The chart demonstrates that certain cannabinoids elicit similar IC_50_ values between specific types of immune cells while other display a differential potency. (D) IC_50_ values of the nonacidic version of the top 5 most potent cannabinoids represented in a radar chart format to highlight the difference in potency between these compounds and their acidic counterparts in C as well as across the 5 immune cell lines.

Inflammation or tissue damage is a multicellular process that involves various types of infiltrating or resident peripheral immune cells at the site of inflammation or damage as well as at other pain-relevant sites containing sensory neurons. Understanding how individual cannabinoids affect different types of immune cells is key to determining which compounds can be used alone or in combination to achieve the desired effect at the site of inflammation and/or pain. To assess the differential effect of cannabinoids on various cell types, we carried out HTS of all 22 compounds on SOCE in monocytes (U937 and THP-1), mast cells (Luva and RBL-2H3) as well as in a model of epithelial cells (HEK-293), all in comparison to our results in T cells (Jurkat). The IC_50_ values across the cell lines are summarized in Figure 11A. Not surprisingly, the cannabinoids inhibited SOCE at different degrees of potency within the same cell line, just as we observed initially in Jurkat cells. However, we also observed that the potency of individual cannabinoids varied depending on the cell type. The cannabinoids are listed (Figure 11A) in the order of their overall potency across cell lines. The top 5 most potent are CBGA, cannabigerovarinic acid (CBGVA), CBDA, THCA, and cannabinolic acid (CBNA) (Figure 11A). These acidic cannabinoids are exclusively found in raw plant material as illustrated in the biosynthesis pathway of cannabinoids in Figure 11B. This biosynthesis pathway of cannabinoids begins with CBGA and CBGVA being the major biosynthetic precursors. The nonacidic compounds, including the major cannabinoids CBD and THC, are mostly found in heated and aged plant material. When comparing the inhibitory effect of the acidic cannabinoids on SOCE to that of their decarboxylated counterparts, we found a significant drop of potency in the latter (Figure 11C and D). The radar plots (Figure 11C and D) showcase the tendency for particular cannabinoids to have higher potency in specific cell types indicating that the mechanism of action of these cannabinoids may be cell type specific. This suggests that cannabinoids can be used alone or in combination for targeted intervention against particular cell types.

## Discussion

Previous research has indicated that various cannabinoids may have anti-inflammatory and antinociceptive properties. While many studies have focused on the treatment of pain through the modulation of nociceptors, specialized peripheral sensory neurons, in the skin that respond to extreme pressure and temperature as well as toxic and noxious molecules,^3,23,24,33^ relatively little is known about the mechanisms of cannabinoids on Ca^2+^ mobilization and signaling in the context of inflammation and pain. There is considerable evidence that SOCE and CRAC channels may be involved in nociceptive, inflammatory, and neuropathic pain mechanisms.^34–36^ In this study, we comprehensively evaluated the efficacy of various cannabinoids against SOCE in pursuit of potential anti-inflammatory and analgesic candidates. It is established that Ca^2+^ signaling mechanisms are responsible for eliciting immune cell activation and subsequent cytokine production, with the major Ca^2+^ influx pathway inducing this response to be SOCE. Hence, SOCE was the key upstream event and favorable readout in our assays to screen for inhibitors that target the pro-inflammatory cascade in immune cells. Our results clearly demonstrate that several cannabinoids are effective in reducing Ca^2+^ mobilization through the inhibition of SOCE. Moreover, we demonstrate that certain minor cannabinoids displayed a higher potency against SOCE than the widely studied major cannabinoids (CBD and THC). We further demonstrate that the carboxylic acid derivatives of the pure cannabinoids elicit a greater potency for inhibiting SOCE than their decarboxylated counterparts indicating a possible SAR. This observation is in accordance with previous studies indicating these carboxylic acid-containing cannabinoid species possess increased efficacy in inhibiting the production of anti-inflammatory mediators.^37,38^

Our results identify CBGA as the most potent cannabinoid in blocking SOCE with an IC_50_ of 530 n m and I_CRAC_ with an IC_50_ of 1.3 μm. The small difference in IC_50_ between SOCE and I_CRAC_ measurements may be attributed to the differences in sensitivity of the two techniques or a nonlinear relationship between CRAC channel inhibition and free cystosolic Ca^2+^ levels. It is also possible that CBGA may be affecting other regulatory components of SOCE such as, for example, CRACR2A,^39^ resulting in stronger suppression of Ca^2+^ entry than the direct suppression of CRAC currents alone. It is possible that CBGA could additionally affect other ion channels that regulate the membrane potential (eg, K^+^ or Cl^–^ channels) and affect the driving force for Ca^2+^ entry. Another example of a regulatory mechanism of SOCE and a possible target of CBGA may be the enzymatic activity of the transient receptor potential melastatin 7 (TRPM7).^40,41^ TRPM7 is an ion channel capable of permeating divalent cations and contains an α-kinase domain.^40^ Previous studies demonstrated a link between TRPM7 kinase activity with SOCE. Faouzi et al. demonstrated a significant reduction in SOCE in TRPM7-deficient DT40 (chicken B lymphocytes) cells.^42^ This reduction was rescued by overexpression with hTRPM7-WT construct but not by the kinase dead mutant (hTRPM7-K1648R) or the kinase deleted mutant (TRPM7-ΔKinase), suggesting the significance of TRPM7 kinase activity in modulating SOCE.^42^ Additional studies further support the involvement of the TRPM7 kinase domain with SOCE. Matsushita et al. have shown that in HEK293 cells, the overexpression of TRPM7-WT but not TRPM7-kinase resulted in increased Tg-induced SOCE.^43^ Others have shown TRPM7 kinase-dead murine T cells exhibit significantly decreased Ca^2+^ signals upon stimulation with anti-CD3/CD28 antibodies and a reduced cyclopiazonic acid (CPA)-induced SOCE.^44,45^ We also show that CBGA does not block I_CRAC_ when applied intracellularly and acts only when applied extracellularly. While this would suggest an external or plasma membrane-delimited site of action on CRAC channels, it does not completely rule out the involvement of regulatory proteins. Whether the inhibition of SOCE is due to direct CRAC channel inhibition and/or is caused by indirect suppression via TRPM7 or auxiliary channels and regulatory proteins awaits exploration in future studies.

Our data also demonstrate that CBGA possesses high affinity to BSA, rendering it biologically inactive long-term in in vitro experiments to elicit its inhibitory effects on SOCE and subsequent T cell cytokine production. These results are in line with previous studies demonstrating the sequestering action of serum mainly due to three long-chain fatty acid binding sites located on BSA, resulting in an inverse relationship of cannabinoid activity with serum concentrations in cell viability assays.^29,30,46,47^ This phenomenon of cannabinoid sequestration by BSA may account for loss of efficacy associated with long-term experiments in which standard tissue culture medium is supplemented with FBS. However, for in vivo studies, plasma proteins and serum albumins play a vital role in solubility and transportation of drugs and assessing CBGA bioavailability in vivo can be accomplished by performing spectroscopic, molecular docking, pharmacokinetic, and pharmacodynamic studies, all common practices in drug development that provide valuable information for the design of efficacy studies.^48–51^

Several studies have successfully used cannabinoids or their derivatives, including CBG and CBGA, in animal models, suggesting that reasonable bioavailability of these compounds is achievable in vivo.^31,32^ Acidic cannabinoids CBDA, THCA, CBGA, CBGVA, cannabichromenolic acid (CBCA), and cannabidivarinolic acid (CBDVA) have shown a rapid absorption into the plasma within 15–45 min. CBGA for example reaches a high maximal concentration of 176.14 ± 21.1 µm in murine plasma with a single intraperitoneal (i.p.) injection of just 10 mg/kg, which is above the concentration needed to achieve the inhibition of SOCE.^31,52^ Anderson et al. also showed that these acidic cannabinoids are absorbed into brain tissue and the type of vehicle can change the brain-plasma ratios, as they exhibit poor brain penetration in an oil vehicle as opposed to a Tween-based vehicle. This suggests that our lead compound, CBGA, can be used to target peripheral inflammation as well as inflammation in the central nervous system. Further studies would be required to measure the bound and unbound cannabinoids and assess how different routes of administration can affect the pharmacokinetic and tissue distribution of these compounds. A better understanding of the pharmacology of these compounds would facilitate the design of more effective in vivo studies and clinical testing of cannabinoids against inflammation.^31,32^

In addition to demonstrating the SOCE inhibitory effects of pure CBGA, we investigated whether these effects on SOCE by CBGA could be altered with the introduction of a second cannabinoid. The majority of *Cannabis* research has been performed on the major cannabinoids, but evidence supports the notion of minor cannabinoids, terpenes, lignans, and flavonoids may contribute to a synergistic interaction commonly reffered to as the “entourage effect.”^53,54^ Previous studies have demonstrated that *Cannabis*-derived extracts present a better efficacy than pure individual cannabinoids, with extracts rich in CBD and/or THC containing small concentrations of minor cannabinoids, such as CBG and THCA.^55^ Moreover, Blasco-Benito et al. investigated this entourage effect by comparing pure cannabinoids and botanical extracts with corresponding concentrations of terpenes with standard chemotherapeutic drugs used in different breast cancer therapies.^52,55^ Interestingly, the authors found that in all cases the botanical extract outperformed the pure cannabinoids in cell cultures but not in vivo and both the botanical extract and pure cannabinoids had no impact on standard chemotherapeutic therapies.^55^ Our study investigated the potential entourage effect between our most potent cannabinoid, CBGA, in combination with one additional cannabinoid. While our results showed some supra- or subadditive effects on SOCE, these were rather moderate and generally did not exceed 10%. A possible explanation for this could be that experiments by Blasco-Benito et al. compared a botanical extract and pure cannabinoids with the lone similarity being the concentration of THC, suggesting that an unknown combination of phytochemicals of considerable complexity may be at play.^55^ Furthermore, our study of the entourage effect combines our lead cannabinoid, CBGA, with one additional cannabinoid and does not take into account the possibility of other phytochemicals such as terpenes, lignans, and flavonoids contributing to the biological activity. This could explain the lack of large “entourage effects” between different cannabinoids in our isobolographic assays. We favor the view that entourage effects may not necessarily manifest themselves as targeting a single mechanism such as SOCE, but more likely be caused by the complementary effects of various phytochemicals on multiple mechanisms or targets within a given cell or even across multiple cell types contributing to chronic inflammation.

Indeed, our results also highlight the similarities and differences between the panel of pure cannabinoids screened against SOCE within several different immunological cell lines. We thoroughly screened each cannabinoid at different doses to determine IC_50_ values in the various immune cell types, all of which exhibited the presence of SOCE. The results show varying degrees of IC_50_ values for different cannabinoids within a single cell line as well as across cell lines, suggesting significant diversity in molecular targets that underlie or modulate SOCE in these cell types. Previous research has demonstrated that SOCE regulation involves not only complexities in the composition of primary SOCE molecules itself (STIM1 and STIM2 acting as ER Ca^2+^ sensors and Orai1/2/3 as homo- or even heteromeric CRAC channel units), but also additional cell-specific environments and multiple modulatory mechanisms. These mechanisms include post translational modifications of different targets critical for SOCE such as phosphorylation, ubiquitination, and glycosylation of Orai and STIM isoforms, in addition to other interacting proteins possibly involved in recruitment and translocation of Orai1 and STIM1 to ER/PM junctions.^56–64^ As to which molecular target or combination of targets these cannabinoids may be modulating has yet to be determined.

From these data, it is also interesting to note that the pure cannabinoids can be grouped by their potency against specific cell types. The heat map table (Figure 11A) demonstrates that cannabinoids containing carboxylic acid functional groups possess the highest potency for SOCE inhibition in T cells. The radar chart (Figure 11C) shows that certain cannabinoids have similar IC_50_ values within specific cell types. It is tempting to speculate that the mechanism of action by cannabinoids in these specific cell types could be similar. Future experiments should take into consideration not only the potency of each cannabinoid but the specific cell type that the cannabinoid exerts its activity against. Thus, certain cannabinoids appear to have very specific activities on SOCE in different cell types: (1) CBGA appears to be relatively potent in most cell types, but highly potent in T cells and human mast cells. (2) CBNA and CBCA are highly specific and potent in T cells and relatively ineffective in the other cell types tested. (3) THCA, THC, and CBDV are highly potent and selective in human mast cells, but not very effective in all other cell types. This could be used to develop targeted therapeutic uses for diseases in which these cell types play important roles.

In summary, we have shown the SOCE inhibitory activity of cannabinoids through a combination of methods including HTS assays, T cell activation assays, cytokine production, and electrophysiological mesurements. We have also discovered a previously unrecognized mechanism of action due to inhibition of CRAC channels and the resulting suppression of SOCE. This SOCE inhibitory activity could be instrumental in developing alternative therapies for T cell-mediated inflammation. Importantly, we show that carboxylated cannabinoids elicit a more potent inhibition of SOCE compared to the decarboxylated forms, which in general lack psychoactivity and may therefore be more desirable as therapeutics. Additionally, we show the differences in potency between cannabinoids within the same cell line as well as across cell lines and that the entourage effect could be influenced by not only the specific cannabinoids but also the specific cell types being targeted. Taken together, these data illustrate the complexity and promising potential of cannabinoid and *Cannabis*-based therapies against SOCE-mediated inflammation.

## Data Availability

The data underlying this article will be shared on reasonable request to the corresponding author.
